# Synthesis and Characterisation of Reduced Graphene Oxide/Bismuth Composite for Electrodes in Electrochemical Energy Storage Devices

**DOI:** 10.1002/cssc.201601553

**Published:** 2017-01-18

**Authors:** Jiabin Wang, Han Zhang, Michael R. C. Hunt, Alasdair Charles, Jie Tang, Oana Bretcanu, David Walker, Khalil T. Hassan, Yige Sun, Lidija Šiller

**Affiliations:** ^1^ School of Chemical Engineering and Advanced MaterialsBedson BuildingNewcastle UniversityNewcastle upon TyneNE1 7RUUK; ^2^ 1D Nanomaterials GroupNational Institute for Materials Science (NIMS)Sengen 1-2-1TsukubaIbaraki305-0047Japan; ^3^ Centre for Materials Physics, Department of PhysicsDurham UniversityDurhamDH1 3LEUK; ^4^ School of Mechanical EngineeringStephenson BuildingNewcastle UniversityNewcastle upon TyneNE1 7RUUK; ^5^ Department of PhysicsUniversity of WarwickCoventryCV4 7ALUK

**Keywords:** bismuth, composite materials, energy storage, nanotechnology, reduced graphene oxide

## Abstract

A reduced graphene oxide/bismuth (rGO/Bi) composite was synthesized for the first time using a polyol process at a low reaction temperature and with a short reaction time (60 °C and 3 hours, respectively). The as‐prepared sample is structured with 20–50 nm diameter bismuth particles distributed on the rGO sheets. The rGO/Bi composite displays a combination of capacitive and battery‐like charge storage, achieving a specific capacity value of 773 C g^−1^ at a current density of 0.2 A g^−1^ when charged to 1 V. The material not only has good power density but also shows moderate stability in cycling tests with current densities as high as 5 A g^−1^. The relatively high abundance and low price of bismuth make this rGO/Bi material a promising candidate for use in electrode materials in future energy storage devices.

## Introduction

The efficient storage of energy is a key challenge in the adoption of renewable energy sources and the deployment of clean power technologies. In recent years, supercapacitors have been considered as promising candidates for the next generation of energy storage devices.^1^ Compared with batteries, supercapacitors have higher power density and better cycle life.1b, 2 However, their low energy density and small potential window limit their applications.^3^ It has been suggested that this problem may be addressed by hybrid systems that merge the advantages of supercapacitors and batteries,^3^ often termed “supercapatteries”. Such hybrid systems may involve an asymmetric cell structure of a capacitive and a battery electrode^4^ or, as reported here, composite electrodes that display elements of both supercapacitive and battery behaviours.^5^

There have been a number of previous studies in which composites of carbon and metal oxide were synthesized and their electrochemical properties analysed.^6^ However, few studies have been reported on metal and metal composite materials.^7^ Ag/C electrodes prepared through a facile hydrothermal method followed by a calcination step achieved a capacity value of 211 mA h g^−1^,^8^ whereas Ru/mesoporous carbon composites synthesized by a microwave‐assisted method reached a specific capacitance value of 287 F g^−1^.7b Ru/carbon nanocomposites prepared by a polyol process at 170 °C have Ru particles attached to the carbon surface7c and with 60 % Ru loading, this composite achieved a specific capacitance of 549 F g^−1^.7c Some other metal nanoparticles, such as Au and Ag, have also been considered in electrodes and achieved a capacitance of 70 F g^−1^.7d However, the relatively low abundance and high cost of these noble metals limit their commercial applications. With increasing global concern regarding energy saving, environmental protection and CO_2_ emissions, the search for a low‐cost and environmentally friendly material for electrodes in energy storage devices is important.

Bismuth, as one of the post‐transition metals, has a stable +3 oxidation state and can also exist at a+5 oxidation state. Its good electrochemical properties and environmentally friendly nature make bismuth an excellent candidate for use in electrode materials.^9^ Recently, bismuth was reviewed as one of the most extensively studied elements in solid‐state physics because of its electronic properties, such as a long Fermi wavelength (around 30 nm^10^) and high Hall coefficient.10a A particularly attractive feature of bismuth is that, in spite of its heavy metal status, it is considered a safe and non‐toxic material.^11^ Moreover, a large amount of bismuth is produced as a by‐product of the copper and tin refining industry.^11^ All these attributes make bismuth a promising candidate for electrochemical energy storage materials.

Here, we report on a novel material, a reduced graphene oxide/bismuth composite (rGO/Bi). This composite material was prepared by a modified low‐temperature polyol process, in which hydrazine was used as the reducing agent^12^ while ethylene glycol (EG) was used as both solvent and reducing agent. An intermediate complex is formed by EG and the metal ions absorbed on the rGO surface producing nano‐sized particles and preventing aggregation.^13^ Bismuth particles, which are oxidised and reduced during electrochemical cycling, are formed with an approximate lateral size of 20 to 50 nm and attach to the rGO sheets. Assembly of graphene into three‐dimensional structures has the potential of creating electrodes with extremely large (and accessible) specific surface areas coupled with good electrical conductivity, which enables fast electron transfer.^14^ The decoration of such structures with faradaic charge storage materials can create composite electrodes that maximize electrode capacity beyond that offered by the theoretical upper limit of 550 F g^−1^ (550 C g^−1^ at 1 V) in carbon‐based materials.^14^

Two composite materials, similar to that presented herein, were the subject of previous investigation. Wang et al.^15^ investigated the electrochemical charge‐storage behaviour of amorphous carbon–bismuth oxide composites with Bi_2_O_3_ contents of between ∼14 and 33 %, which they incorrectly characterised as pseudocapacitive. It is important to differentiate between the specific capacitance and the specific capacity of an electrode.^16^ The former refers to the capacitance per unit mass and is only applicable to charge storage that is (pseudo)capacitive in nature—i.e., demonstrates an almost rectangular cyclic voltammogram (CV) and linear galvanostatic charge/discharge (GCD) characteristics. Materials displaying non‐capacitive faradaic charge storage (battery materials), which possess peaks in CVs and plateau regions in GCD curves should be characterised in terms of the second quantity, the total charge stored per unit mass. From the GCD data presented by Wang et al.^15^ it is possible to derive a specific capacity for their amorphous carbon/Bi_2_O_3_ composite of ∼333 C g^−1^ at 1 A g^−1^.

The electrochemical behaviour of a rGO/Bi_2_O_3_ composite containing 23.85 wt % Bi_2_O_3_ was also studied.9d Once more, this material was wrongly described as pseudocapacitive, the GCD data showing battery‐like behaviour. From the GCD curve presented in that work it is possible to derive a specific capacity for the rGO/Bi_2_O_3_ composite of 204 C g^−1^ at 1 A g^−1^. Here we report the structure, composition and electrochemical performance of a rGO/Bi composite with a specific capacity of 460 C g^−1^ at 1.2 A g^−1^, which is substantially larger than that of the previously reported materials, and reaches 773 C g^−1^ at 0.2 A g^−1^. We suggest that the improved specific capacity of the composite detailed in this work arises from the excellent electrical conductivity afforded by the rGO backbone, the good electrical contact with the bismuth particles, which are initially deposited in metallic form, and a high utilization of bismuth during charge/discharge, which is related to the microstructure of the composite.

## Results and Discussion

**Figure 1:**
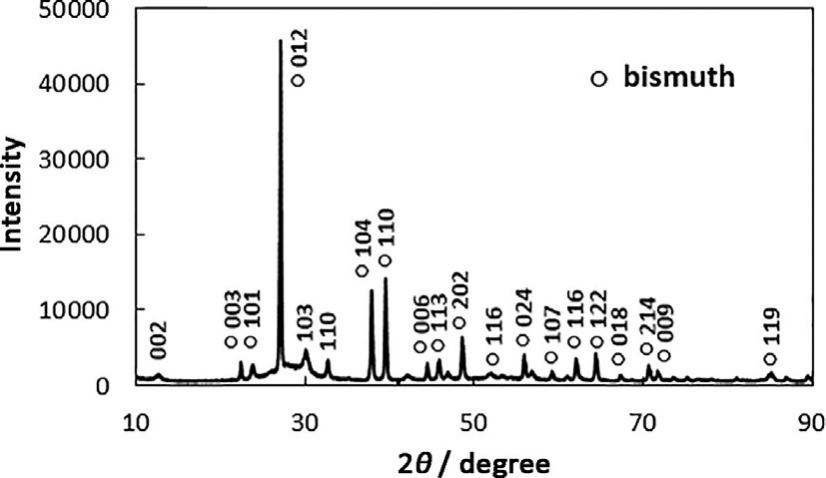
X‐ray powder diffractogram of rGO/Bi.

An X‐ray powder diffractogram (XRD) of the as‐prepared rGO/Bi composite is shown in Figure 1. The strongest three peaks appear at 27.06, 37.80 and 39.46°, which correspond to the (012), (104) and (110) reflections of bismuth, respectively (Natl. Bur. Stand., U.S.), and therefore confirm the dominant presence of bismuth metal on the graphene surface. The weak peak that appears at 12.64° indicates an interlayer spacing of 0.7 nm, which could be related to graphene oxide (GO).^17^ The small hump around 25° is caused by the disordered stacking of layers of rGO.^18^ Peaks with positions at 30.02 and 32.66° cannot be indexed with the crystal structure of bismuth, but are in agreement with the (103) and (110) crystal planes of bismuth subcarbonate (Natl. Bur. Stand., U.S.). Both GO and EG, which were used as starting materials, could be the carbon source for the Bi_2_O_2_CO_3_ observed. The absence of peaks related to bismuth oxides in the diffractogram of the as‐prepared composite indicates that the starting material primarily consists of rGO and metallic bismuth. It has been observed previously that bismuth metal nanostructures, such as nanowires or nanoparticles, readily oxidize when exposed to air at atmospheric pressure.^19^ Metallic bismuth wires typically have an oxide layer ∼1 nm thick after 4 h exposure to air.19b After 48 h exposure, the thickness of the oxide layer is ∼4 nm.19b High temperature hydrogen and ammonia environments were found to reduce the oxide without damaging the bismuth metal after a sufficient amount of time, but the oxide was found to reform in less than 1 min of exposure to air.19c We note that graphene sheets act as impermeable atomic membranes to many gases^20^ and therefore it is likely that the absence of significant bismuth oxidation observed in the as‐prepared materials is related to a retardation of this process though protection of bismuth by rGO.

**Figure 2:**
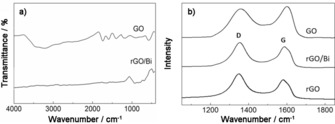
a) FTIR spectra of GO and rGO/Bi, b) Raman spectra of GO, rGO/Bi and rGO.

In the Fourier Transform Infra‐Red (FTIR) spectroscopy data from GO, Figure 2 a, a broad peak is present between 800 to 1400 cm^−1^ which can be assigned to in phase C−C−O stretching (800–1000 cm^−1^), out of phase C−C−O stretching (1000–1260 cm^−1^) and C−O−H bending (1200–1430 cm^−1^) modes.^21^ The peaks observed at around 1600 and 1720 cm^−1^ are attributed to the skeletal vibration from unoxidized graphitic domains and the C=O stretching of unsaturated carbonyl groups, respectively.^21^, ^22^ The broad peak appearing at 3200–3600 cm^−1^ originates from the hydrogen bonded OH stretching vibration.^21^, ^23^ In the FTIR spectrum from rGO/Bi, the peak at 424 cm^−1^ mainly arises from the displacement of oxygen atoms with respect to bismuth causing Bi−O bond elongation.^24^ The peak that appears at 675 cm^−1^ results from Bi−O bonds of different lengths in distorted BiO_6_ units.^25^ The broad peak at around 845 cm^−1^ can be attributed to the antisymmetric stretching of CO_3_ groups.^26^ Compared with the FTIR result from GO, rGO/Bi has fewer peaks in the range from 1200 to 2000 cm^−1^ and from 3200 to 3600 cm^−1^, which indicates the successful removal of oxygen functional groups from the surface of GO.

Raman spectroscopy was used to compare the density of defects in GO, rGO/Bi and rGO (Figure 2 b). Two obvious peaks, which appear at around 1580 cm^−1^ (G band) and 1350 cm^−1^ (D band), were observed in all three materials. The peak at 1580 cm^−1^ is caused by the in‐phase vibration of the sp^2^ graphite lattice whereas the peak at 1350 cm^−1^ results from structural defects and disorder.^27^ The intensity ratio of the D‐ and G‐band peaks (*I*_D_/*I*_G_) changes from 0.90 in GO to 1.17 in rGO/Bi and 1.29 in rGO, indicating a decrease in the average size of the sp^2^ domains. Similar results have been reported in the literature^12^ and explained in terms of the creation of new graphitic domains upon reduction of GO to rGO, which are smaller in size but larger in quantity compared with those in the starting material.

**Figure 3:**
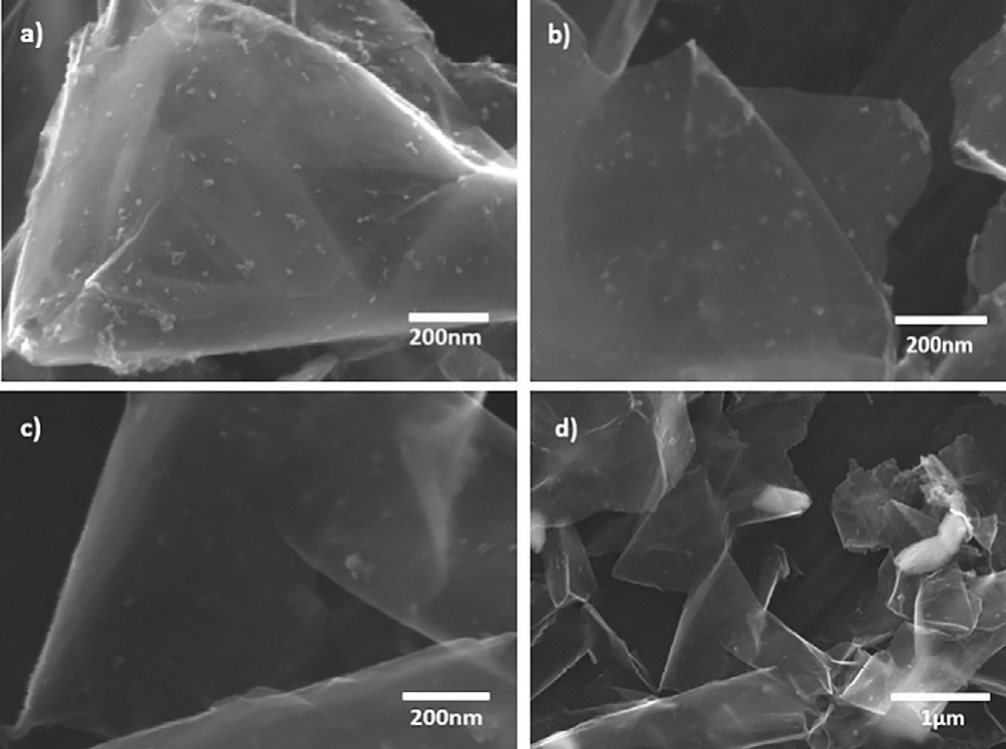
SEM images of the as‐prepared rGO/Bi composite at a) 80 000x, b) 100 000x, c) 100 000x, d) 25 000x magnification.

The layered substance shown in the Scanning Electron Microscopy (SEM) images in Figure 3, with dimensions larger than 1 μm, can be identified as rGO.^28^ Therefore, the particles with lateral sizes in the range of 20 to 50 nm attached to the rGO layers are considered to be bismuth (see also discussion below). In some parts of the rGO/Bi samples bismuth particles are seen to have agglomerated and formed clusters with sizes larger than 500 nm, as shown in Figure 3 d.

**Figure 4:**
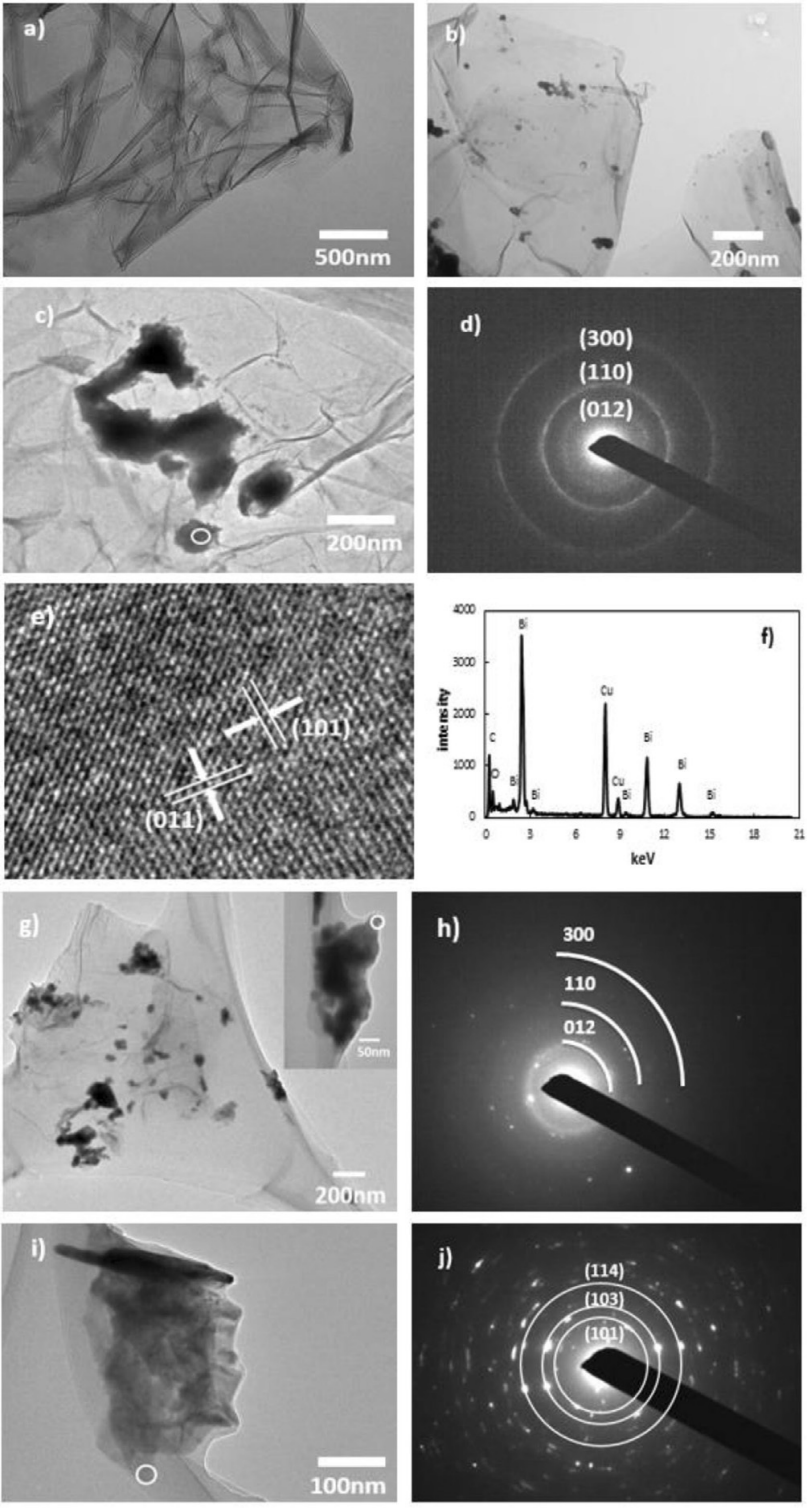
a) TEM image of rGO; b) TEM image of as‐prepared rGO/Bi; c) TEM image of rGO/Bi showing a region containing bismuth agglomerates; d) SAED measured on rGO/Bi; e) crystalline structure observed by HRTEM in as‐prepared rGO/Bi; f) EDS from as‐prepared rGO/Bi; g) TEM image of rGO/Bi after electrochemical cycling; h) SAED after electrochemical cycling showing the presence of metallic bismuth; i) TEM images of rGO/Bi after electrochemical cycling; j) SAED after electrochemical cycling, showing the presence of bismuth subcarbonate.

Transmission Electron Microscopy (TEM) images of rGO and rGO/Bi are shown in Figure 4 a and b, respectively. Agglomeration is observed to occur in isolated regions of the sample, forming bismuth aggregates with lateral sizes larger than 200 nm, as seen in Figure 4 c. A selected area electron diffraction (SAED) pattern (Figure 4 d) of one such particle in Figure 4 c confirms the crystal structure of metallic bismuth. Three rings are observed in this diffraction pattern, which correspond to reflections from the (012), (110) and (300) planes of bismuth metal (Natl.Bur.Stand., U.S.). The SAED pattern is in good agreement with the strong peaks associated with metallic bismuth observed in XRD (Figure 1).

An additional crystalline structure (Figure 4 e) was observed in some locations in the sample. The atomic structure shown in the TEM image could be indexed with the (101) and (011) crystal lattice planes of bismuth subcarbonate. This result confirms the existence of small quantities of bismuth subcarbonate as impurities, again in agreement with the XRD results presented in Figure 1. Energy dispersive X‐ray spectroscopy (EDS) from the rGO/Bi composite (Figure 4 f) displays strong bismuth, carbon and copper peaks. Bismuth peaks originate from bismuth particles and bismuth subcarbonate and the carbon peak could contain contributions from both rGO and Bi_2_O_2_CO_3_. The copper peaks are a result from the copper TEM support grid. The low carbon peak intensity compared with the high bismuth peak intensity suggests that the amount of bismuth subcarbonate is not great. Figure 4 g and i show TEM images of the rGO/Bi composite after cycling. Agglomeration is observed to occur, forming particles with sizes from 100 to 200 nm. Both bismuth (Figure 4 h) and bismuth subcarbonate (Figure 4 j) were observed in the SAED patterns obtained after electrochemical cycling.

**Figure 5:**
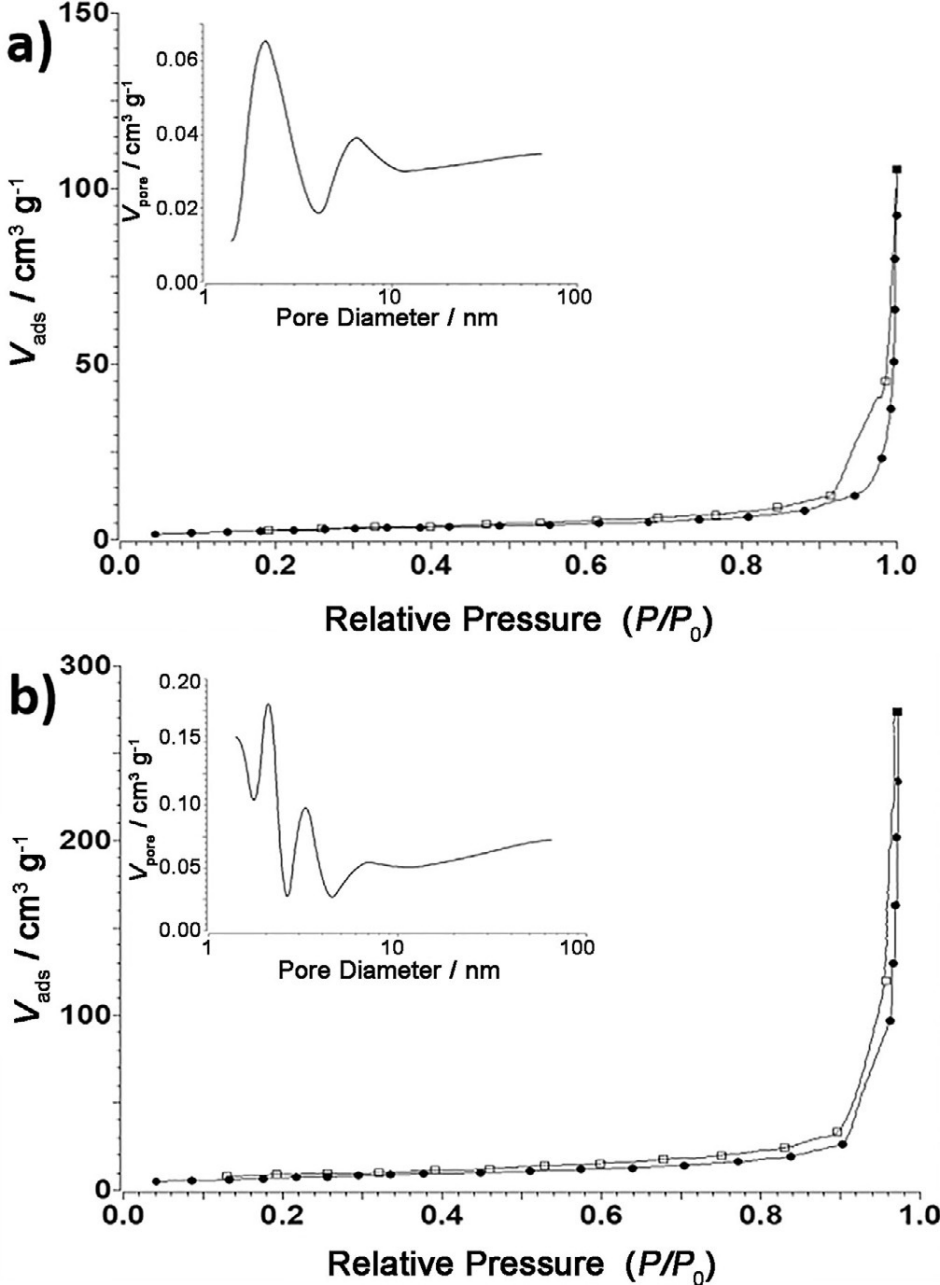
Nitrogen adsorption‐desorption isotherms of a) rGO/Bi and b) rGO.

The microstructure and pore‐size distribution of rGO and rGO/Bi were determined from N_2_ adsorption–desorption isotherms, Figure 5 a and b, respectively. Both isotherms can be classified as type I isotherms for microporous solids.^29^ The as‐synthesized rGO/Bi is found to have a specific surface area of 10.55 m^2^ g^−1^ with pore‐size diameters in the range from 2–8 nm whereas the rGO has a specific surface area of 23 m^2^ g^−1^ with pore‐size diameters of 1–3 nm. Compared with rGO, the rGO/Bi composite has a larger pore size, which may originate from the insertion of bismuth nanoparticles into the material and can facilitate more ready penetration of ions into the composite electrode, increasing surface accessibility. Given that approximately half the weight of the rGO/Bi composite consists of rGO the reduction in specific surface area by a factor of ∼2 suggests that the incorporation of bismuth has not significantly changed the total surface area offered by the rGO component.

**Figure 6:**
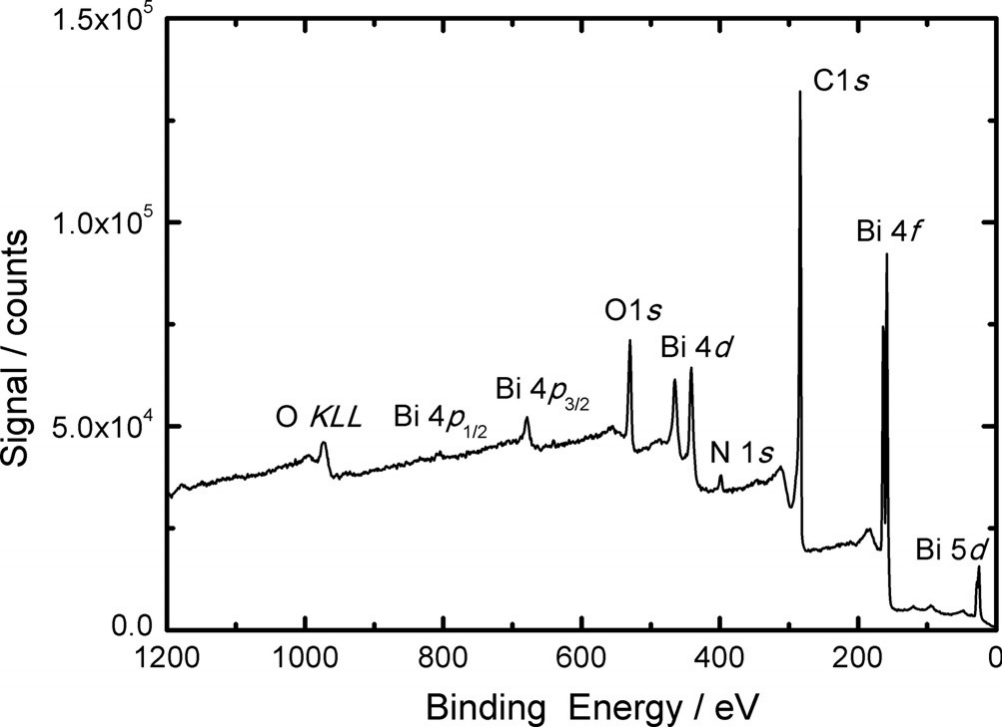
XP survey spectrum obtained from 27 month‐old rGO/Bi composite.

X‐ray photoelectron spectroscopy (XPS) was performed on rGO/Bi composites 27 months after fabrication. Figure 6 shows a survey spectrum obtained from the rGO/Bi composite. There are strong peaks associated with bismuth, oxygen and carbon. A small signal from nitrogen is also present corresponding to a concentration of <2 at % which, in the absence of any signal from Bi(NO_3_)_3_ (see below) is likely to originate from nitrogen inclusion in the rGO resulting from hydrazine treatment, as previously observed by Park et al.^30^ No other elements can be detected.

**Figure 7:**
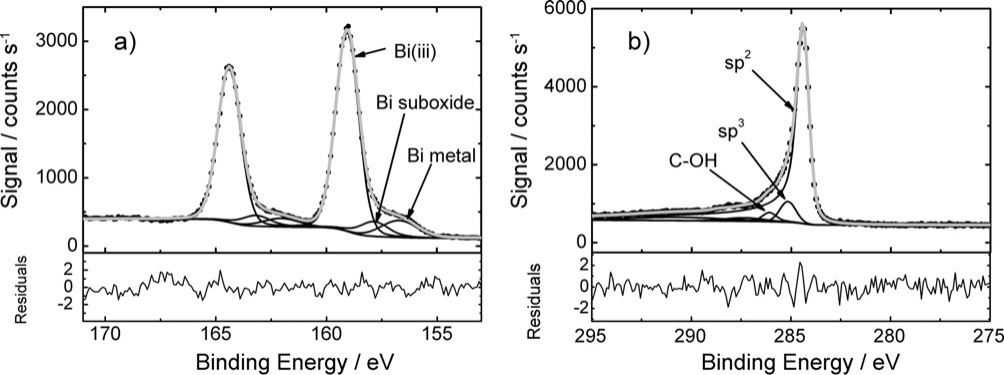
a) Top panel: Bi 4f XP spectrum of the rGO/Bi composite and associated fit. The Bi 4f_7/2_ components are associated with metallic bismuth, Bi suboxide and Bi in the +3 oxidation state (Bi^III^). Bottom panel: Fit residuals in units of standard deviation of the data. b) Top panel: C 1s XP spectrum of the rGO/Bi composite and associated fit. The three largest fit components, associated with sp^2^, sp^3^ carbon and C−OH are labelled. Bottom panel: Fit residuals in units of standard deviation of the data. In both spectra black dots represent the experimental data, the grey line the fit to the spectrum and the black lines the individual fit components and Shirley background.

A high resolution XP spectrum of the Bi 4f lines is presented in Figure 7 a along with the associated fit. Three components are necessary to fit the spectrum, the strongest a doublet with the 4f_7/2_ component at 159.05±0.04 eV and a 4f_5/2_ component 164.38±0.04 eV that corresponds to Bi in the +3 oxidation state in Bi_2_O_2_CO_3_ 31 and Bi_2_O_3_.^32^ The doublet located at 156.70±0.04 eV (4f_7/2_) and 162.03±0.04 eV (4f_5/2_) is due to metallic bismuth,^33^ whilst the third doublet, located at 157.8±0.1 eV (4f_7/2_) and 163.1±0.1 eV (4f_5/2_), which must be included to ensure an appropriate fit, can be attributed to bismuth suboxides, such as BiO.^34^ There is no evidence for Bi 4f components associated with residual Bi(NO_3_)_3_ 33 or Bi in the +5 oxidation state in the XP spectrum. The relative strength of the Bi^III^‐related doublet in comparison with that of the metal is explained by the surface sensitivity of XPS: using the approach of Tanuma, Powell and Penn^35^ we determine the electron inelastic mean‐free path for the Bi 4f lines to be ∼3 nm. Hence, a thin oxide layer present on bismuth particles at the surface of the composite would be expected to dominate the XPS signal. Indeed, the presence of a 4f component associated with metallic bismuth demonstrates that the surface oxide layer is no more than a few nanometres in thickness.

A high resolution XP spectrum of the C 1*s* region is shown in Figure 7 b. The signal is dominated by an asymmetric graphitic line (Doniach‐Šunjić line‐shape, *α*=0.14) with a binding energy of 284.40±0.05 eV, consistent with graphitic materials. Small peaks (<5 % of total C 1*s* intensity) associated with C−OH, C=O and O=C−OH are located at 286.1±0.5, 287.3±0.5 and 288.7±0.5 eV binding energy, respectively, reflecting residual oxygen containing groups on the rGO surface.^36^ The fit component associated with C−OH is the largest of these, consistent with previous observations that residual −OH groups are the most prevalent oxygen containing groups in rGO after hydrazine treatment^36^ (although there may also be a contribution to this component from carbon bound to nitrogen^12^). To obtain a good fit, it was also necessary to include a minor peak (<10 % of total C 1*s* intensity) at 285.2±0.2 eV, which has previously been associated with sp^3^‐hybridised defects within nanostructured carbons^37^ suggesting that residual disorder remains in the rGO when oxygen‐containing groups are removed.

The composition of the rGO/Bi composite was determined from the XP spectra by standard approaches^38^ using photoelectron cross‐sections calculated by Yeh and Lindau^39^ and inelastic mean‐free paths determined as above.^35^ The composite was found to contain carbon, oxygen and bismuth in the (atomic) ratio 0.78:0.18:0.03 (with an estimated error of ±0.02 for each species).

**Figure 8:**
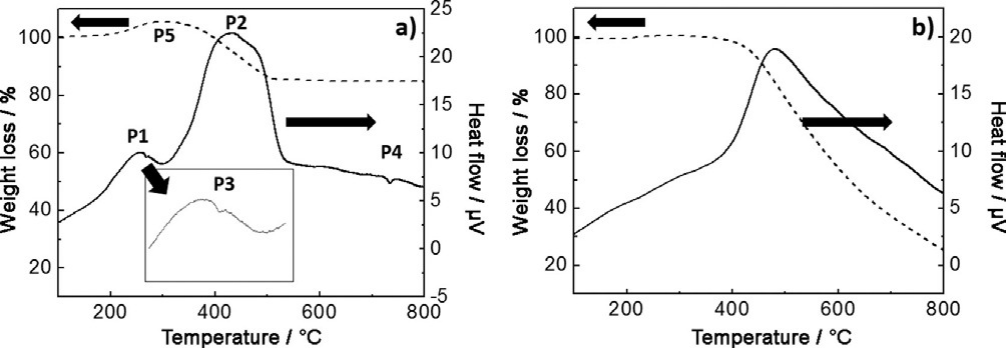
DTA and TGA curves of a) rGO/Bi and b) rGO.

Differential thermal analysis (DTA) and thermo‐gravimetric analysis (TGA) curves of rGO/Bi and rGO are presented in Figure 8. The DTA data from the rGO/Bi composite (Figure 8 a) show two broad exothermic peaks, P1 and P2, and two very small endothermic peaks, P3 and P4. P1 can be attributed to the adsorption of oxygen at the surface of rGO in the presence of bismuth at low temperatures (175–250 °C). The small endothermic peak P3, at about 275 °C, is associated with the melting of metallic bismuth. The exothermic peak P2 is very broad and represents an overlap of different exothermic processes: oxidation of bismuth between 325–375 °C which involves a mass increase of 2–3 wt % and carbon combustion between 355–525 °C, accompanied by a mass loss of 18–20 wt %. The small endothermic peak, P4, at 730 °C has no mass variation associated with it and probably corresponds to melting of bismuth oxide, with the melting peak shifted to low temperatures because of the nanometer‐scale dimensions of the particles. The broad peak labelled P5 in the TGA data from rGO/Bi, showing a mass increase of around 5 wt %, probably corresponds to the combined effects of the processes described by the exothermic peak P1 and part of P2 in the DTA curve, attributed to bismuth oxidation.

The DTA curve of rGO powder (Figure 8 b) exhibits only one broad exothermic peak with an onset temperature of about 400 °C. This exothermic behaviour is attributed to carbon combustion in air and takes place with a mass loss of 74 wt %. In the absence of bismuth the rGO combustion peak is shifted to higher temperatures. Over the temperature 450 to 800 °C rGO/Bi has a smaller weight loss compared with rGO. This might be because by adding the bismuth nanoparticles, the rGO/Bi has better graphitization and de‐oxygenation with enhanced van der Waals interactions between layers.^40^

Based on the 5.13 % weight gain (peak P5), bismuth and rGO have a weight ratio of 0.44:0.56. This is in good agreement with the atomic ratio given by XPS (bismuth and carbon were found by XPS to have an atomic ratio of 0.03:0.78, as discussed above, which corresponds to a weight ratio of 0.4:0.6).

**Figure 9:**
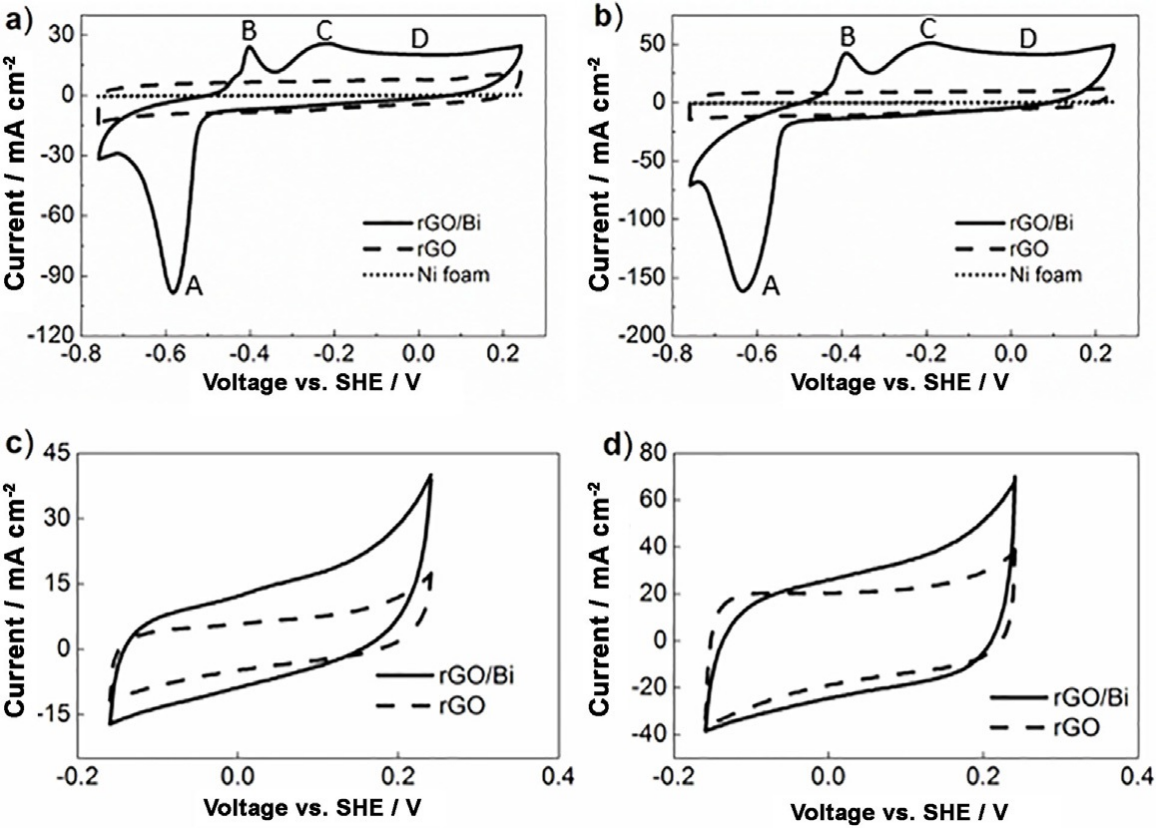
Typical CV results of rGO/Bi, rGO and Ni foam within different voltage range at scan rates of a) 20 mV s^−1^, b) 50 mV s^−^,^1^ c) 20 mV s^−1^, d) 50 mV s^−1^.

Electrochemical properties of the as‐prepared rGO/Bi, rGO and Ni foam were analysed by CV under different scanning rates, as shown in Figure 9. CV measured at different scanning rates, 20 and 50 mV s^−1^, presented similar shaped curves. Voltages from 0.2 to −0.8 V versus standard hydrogen electrode (SHE) were applied. Three clear peaks (A, B and C) and a small plateau (D) were observed in the CV experiments. Peak A, which appears at around −0.7 V, is associated with the reduction of bismuth from the +3 oxidation state to the metallic state (0 oxidation state).^41^ Peaks B and C, which appear at −0.5 and −0.3 V, represent the formation of BiO_2_^−^ and Bi(OH)_3_ during the oxidation of bismuth from metal to the +3 oxidation state.^41^ The surface layer of bismuth was partially dissolved in the KOH electrolyte and forms BiO_2_^−^ in the first reduction reaction.^42^ It is possible that the plateau D may be due to the oxidation of un‐transformed bismuth.^41^ A previous study has shown that this plateau becomes dominant in bismuth films as the film thickness is reduced.^41^ The plateau has only been observed in thin bismuth (metal) films with highly rough surfaces.^41^ In Ref. 41 it is suggested that very high bismuth oxidation states of +4 or +5 might occur because of the hypothetical formation of gel like electrolyte (when bismuth metal is rough) and these oxidation states are responsible for the observed plateau through faradaic processes. However, CV curves of rGO/Bi in the range from −0.2 to 0.24 V (Figure 9 c and d) display a rectangular shape almost identical to that of rGO when scaled for the rGO mass content. It is therefore more likely that the constant capacitance value in this potential window indicates that, over this range of potential, the rGO/Bi displays an electrical double layer (EDL) capacitance originating primarily from the rGO component in the composite and that the composite electrode therefore demonstrates both supercapactive and battery chracteristics.

During the oxidation and reduction processes intermediate products, which include Bi(OH)_3_, BiOOH and BiO_2_^−^, may be formed as follows:^41^, ^43^

Peak A 1

$$BiO_{2}^{-}+\;e^{-}\to \;BiO_{2}^{2-}BiO_{2}^{2-}+3H_{2}O\;\to 2BiO_{2}^{-}+4OH^{-}+{Bi}^{\left(0\right)}\;{Bi}^{\left(0\right)}\;\to \;{Bi}_{\left(metal\right)}$$

Peaks B and C, 2

$${Bi}^{\left(0\right)}+{OH}^{-}\to {BiO}_{2}^{-}+Bi(OH{)}_{3}+H_{2}OBi(OH{)}_{3}\;\to BiOOH+\;H_{2}OBiOOH+\;{OH}^{-}\;\to \;{BiO}_{2}^{-}+\;H_{2}$$

CVs of pure Ni foam and rGO were also measured and are presented in Figure 9 a and b for comparison. The CV of rGO measured at both 20 and 50 mV s^−1^ show rectangular shapes without any noticeable peaks, which indicates that the capacitance of rGO only arises from the EDL capacitance.^44^

**Figure 10:**
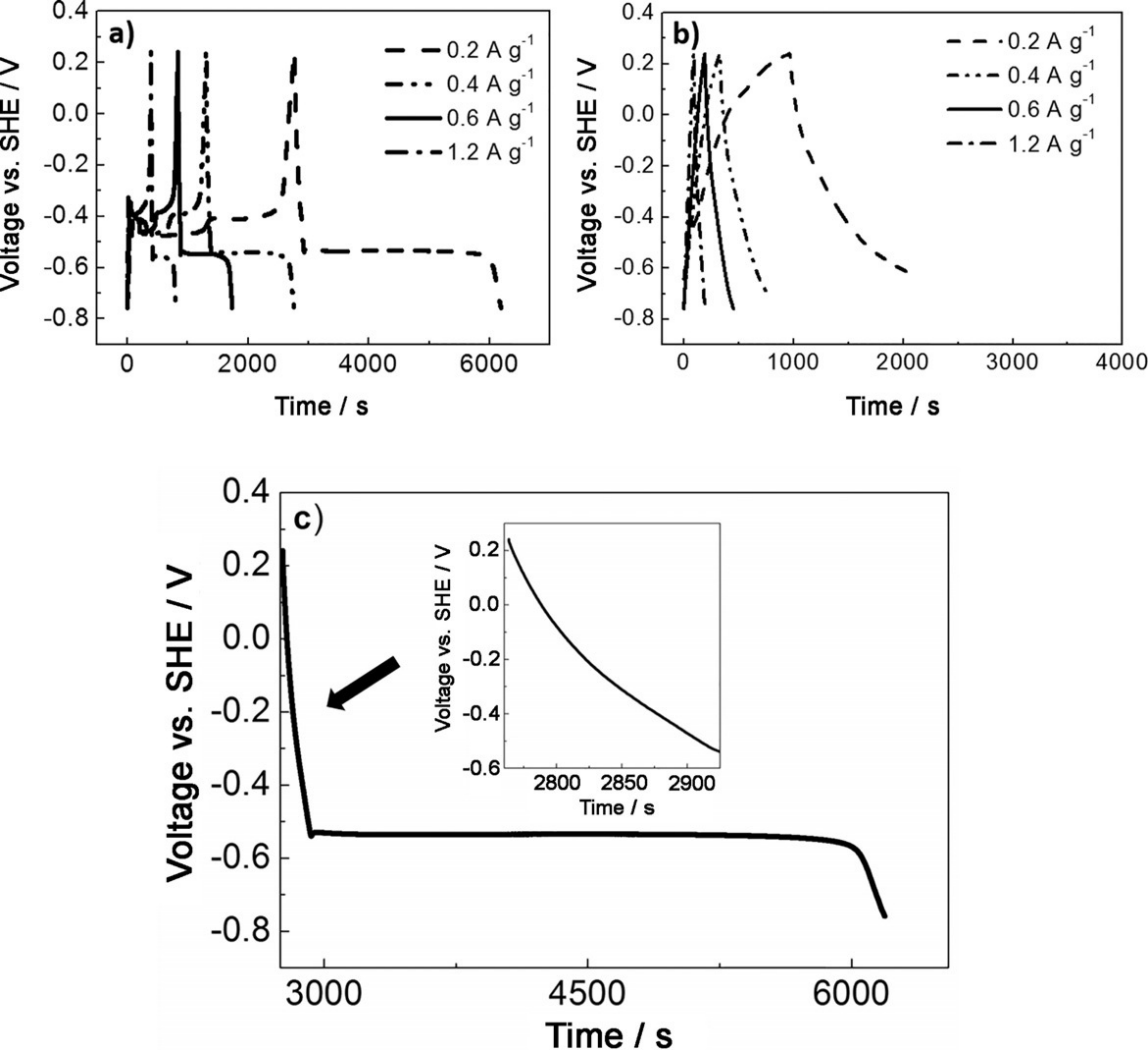
Typical charge/discharge behaviours of a) rGO/Bi at different current densities, b) rGO at different current densities, c) enlarged discharge curve of rGO/Bi at 0.2 A g^−1^.

Chronopotentiometry was used to study the charge/discharge behaviours of the as‐prepared rGO/Bi and rGO materials. The GCD curves were measured at different current densities, ranging from 0.2 to 1.2 A g^−1^ (Figure 10). The GCD curves of rGO/Bi show similar behaviour at different current densities. In the enlarged discharge curve of rGO/Bi, as in Figure 10 c, both slope and plateau were observed.

The quasi‐linear behaviour at the beginning of the discharge curve indicates a contribution from capacitor‐like behaviour. This originates from charge stored electrostatically16b on the surface of rGO, as described above. The plateau indicates material undergoing a phase transformation during the redox reaction,6b as described by peak A in Figure 9 a. The charge/discharge characteristics of rGO, presented in Figure 10 b, do not contain any obvious peaks, in agreement with the CV results of Figure 9 b. Since the energy storage mechanism of rGO/Bi includes a significant non‐capacitive faradaic or battery‐like contribution, the appropriate way to measure the amount of charge stored in the electrode is the specific capacity (*C*_s_) using *C_s_=I* Δ *t/m*,^3^, ^45^ where *C*_s_ is the specific capacity (C g^−1^), *i*/*m* is the current density employed in the measurement (A g^−1^) and Δ*t* is the discharge time in seconds.^45^

**Figure 11:**
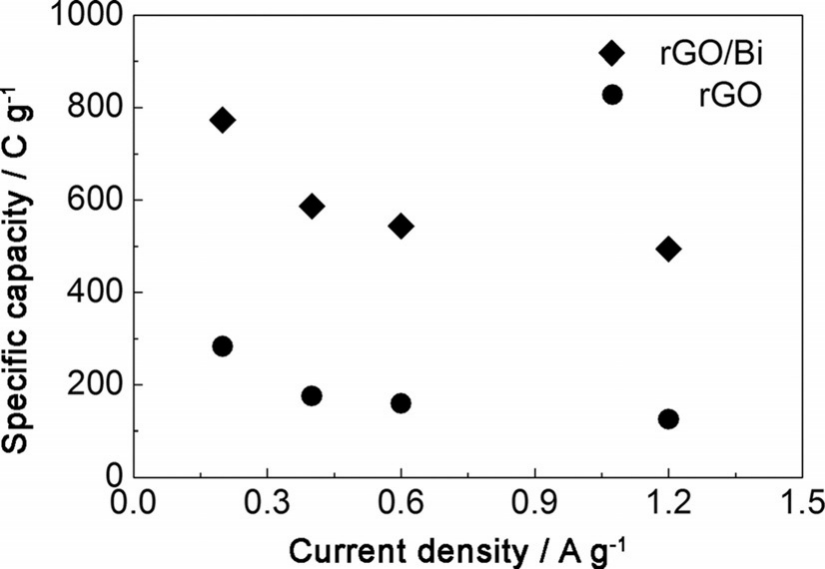
Specific capacity of rGO/Bi and rGO calculated from charge/discharge curves.

Figure 11 shows the specific capacity of the rGO/Bi composites calculated from the GCD curves. Composite samples achieved a specific capacity value as high as 773 C g^−1^ at a current density of 0.2 A g^−1^. The specific capacity is seen to decrease as the current density increases, which can be attributed to incomplete utilization of the active material at high current densities.9c When a high current density is used, the redox reaction only occurs at the surface of active materials.9b However, the rate at which the specific capacity drops decreases with increasing current density, indicating that the electrode material can still show good capacity even at high current density. When the current density reaches the range of 0.4–1.2 A g^−1^, the specific capacity maintains almost a constant value, in the range of 587–494 C g^−1^. The specific capacitance of the pure rGO was found to be 283 F g^−1^ at a current density of 0.2 Ag^−1^, which is comparable to the value of 205 F g^−1^ found for gas‐phase reduced rGO.^46^ At a current density of 1.2 A g^−1^, the specific capacitance of rGO was found to decrease to 125 F g^−1^, which, at a potential of 1 V, stores approximately a quarter of the capacity value found for the rGO/Bi material.

From the bismuth content of the rGO/Bi composite it is possible to calculate the maximum theoretical contribution to the total specific capacity of the electrode from this component of the material. The specific capacity associated with oxidation of bismuth is 1385 C g^−1^ resulting in a contribution to the electrode material of 610±20 C g^−1^ (170±6 mA h g^−1^). If the specific capacitance of the rGO in the composite is unaltered, we would therefore expect a contribution to the specific capacity of the electrode of 160±3 C g^−1^ when the voltage range of the galvanic discharge curve is 1 V (as used in our experiments). Hence, we would expect a theoretical specific capacity of 770±20 C g^−1^ for the composite over a potential of 1 V if all the bismuth present participates in electrochemical storage, which is remarkably close to the 773 C g^−1^ measured at a discharge current of 0.2 A g^−1^. This result suggests high accessibility of the bismuth within the rGO/Bi composite, reflecting the larger pore size of the composite material, compared with rGO, as described above.

**Figure 12:**
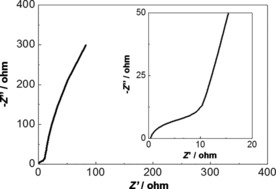
Nyquist plot for the rGO/Bi composite. The inset provides an enlarged view of the high frequency region.

Electrochemical impedance spectroscopy (EIS) was performed on the rGO/Bi composite in a frequency range from 10 mHz to 10 kHz using an alternating current (AC) amplitude of 5 mV, Figure 12. A semicircle is observed in the high frequency region of the plot (inset of Figure 12) corresponding to the faradaic processes, while the linear part in the low frequency region corresponds to ion diffusion capacitive behaviours.^47^ The solution, or series, resistance (*R*_s_) and the charge transfer resistance (*R*_ct_) can be estimated from the intercepts of the semicircle on the real axis,^15^ which are 0.3 and 10 Ω, respectively. The low value of *R*_s_, which originates from the resistance of the electrolyte and the internal resistance of the electrode,^48^ indicates that the rGO composite is highly conductive, facilitating rapid charge transport. The small value of *R*_ct_ suggests that the electroactive bismuth particles are well coupled to the rGO support, which might arise from the metallic nature of the particles in the as‐prepared material.

**Figure 13:**
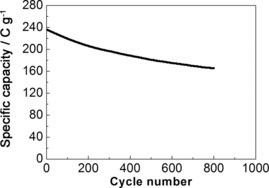
Life‐cycle test of rGO/Bi composite under 5 A g^−1^ current density.

Cycling performance was determined by repeating the charge/discharge test 800 times at a current density of 5 A g^−1^ (Figure 13). This sample achieves a specific capacity of 235 C g^−1^ at the start of cycling, which gradually decreases to 175 C g^−1^ after 800 cycles. Hence, 74.5 % of the specific capacity was maintained after 800 cycles. The gradual decrease of capacity during cycling may be owed to degradation of the active material, bismuth.^49^ In addition, the relatively faster decrease in the capacity of the rGO/Bi composite during the first 200 cycles and slower decrease over the following cycles indicates that structural changes may also have taken place during the charge/discharge cycles, such as the agglomeration observed in the TEM images shown in Figures 4 g and i. After the structure has changed, this sample has a more stable cycling performance, demonstrating a moderate long term electrochemical stability.

## Conclusions

A reduced graphene oxide/bismuth (rGO/Bi) composite, in which the rGO inhibits atmospheric bismuth oxidation, has been synthesized the first time through a polyol process in which ethylene glycol was used as both the solvent and reducing agent. The low reaction temperature, short reaction time and low cost of starting materials make this synthesis procedure appropriate for large‐scale application. The composite material is found to consist of bismuth nanoparticles with lateral sizes between 20 and 50 nm supported by rGO. The as‐prepared rGO/Bi composites displayed specific capacity values as high as 773 C g^−1^ at a current density of 0.2 A g^−1^. The capacity of the rGO/Bi composite described in this work can be attributed to the excellent accessibility of the bismuth and the efficiency of electrochemical reaction resulting from high electrode conductivity and good contact between the bismuth nanoparticles and rGO. Since the electrochemical behaviour of the composite shows contributions from the electrical double layer capacitance of the rGO and faradaic charge storage associated with bismuth, it is reasonable to describe rGO/Bi as a “supercapattery” material. This material has a moderate stability in cycling tests even at current densities as high as 5 A g^−1^. The excellent electrochemical properties of the rGO/Bi composite, simplicity of production and low cost indicate that this material is a promising candidate as an electrode material in electrochemical energy storage devices.

## Experimental Section

Natural graphite flake (99.8 %) and sulfuric acid (98 %) were purchased from VWR. Analytical reagent grade phosphoric acid (85 %), potassium permanganate (99.0 %), dihydrogen dioxide (50 %), bismuth(III) nitrate pentahydrate (>98 %), nitric acid (69 %), and hydrazine solution (35 wt %) were purchased from Sigma–Aldrich, as was the anhydrous EG (99.8 %). All chemicals were used without further purification.

GO was prepared by a modified Hummers method.^50^ Graphite (3 g) and KMnO_4_ (8 g) were weighed and added into a mixture of H_2_SO_4_ (100 mL) and H_3_PO_3_ (20 mL). This suspension was kept at room temperature for three days while stirring continuously. H_2_O_2_ was added into this mixture until it turned a bright yellow colour. This mixture was washed and filtered using 5 % HCl and followed by deionised (DI) water (18 MΩ cm^−1^ resistivity) for several times until a pH of 7 was achieved. GO was obtained after drying the deposit in an oven at 60 °C overnight. Bismuth nitrate (0.3 mmol) and GO (0.03 g) were dispersed into a mixture of EG (23 mL) and nitric acid (2 mL). The suspension was sonicated to reach a homogeneous dispersion. This suspension was transferred into a round bottom flask. Hydrazine (5 mL) was added into this suspension while stirring vigorously. This reaction was held at 60 °C for 3 hours. The synthesized material was collected in a small sample vial after being washed with DI water several times and dried in air overnight. Undoped rGO was synthesized by the same approach to act as a control.

The samples were characterised by XRD (RINT Rigaku), FTIR (Varian 670‐IR), Raman spectroscopy (HR800UV, Horiba, Jobin Yvon), SEM (XL30 ESEM‐FEG, Philips), TEM (JEM‐2100, JEOL), XPS (Kratos Axis Ultra spectrometer), DTA and TGA (Setaram Labsys Evo). A potentiostat (Bio‐logic Science instruments) was used to analyse the electrochemical behaviour of the composites, using CV and measurement of the charge/discharge behaviours. CV results were used to study the mechanism of the reaction taking place during the faradaic redox reaction of bismuth. A small amount (9 mg) of sample was dispersed in DI water. PTFE (10 mg mL^−1^) was added as a binding agent with a sample to PTFE weight ratio of 9:1. After obtaining a homogeneous suspension by sonication, some drops were applied to a nickel foam substrate used as the current collector, working electrode. A three‐electrode system was used for the electrochemical properties test. 2.48 mg mixture of rGO/Bi and PTFE pressed on Ni foam was used as working electrode. A HgO/Hg electrode was used as the reference electrode. A Pt wire was used as the counter electrode and a KOH (6 m) solution was used as the electrolyte. Current densities are quoted in A g^−1^ as the true surface area of the electrodes is difficult to determine.

Samples for SEM imaging were prepared so that a small amount of a sample was dispersed in absolute ethanol. This mixture was sonicated until a homogeneous suspension was achieved. One drop of the suspension was cast on an SEM sample holder and dried in air.

The surface area of the samples was determined from N_2_ adsorption isotherms using a Surfer system (Thermo Scientific). The samples were pre‐degassed for 4 hours at 10^−2^ torr (1.333 Pa) before analysis. The surface area was calculated by measuring the amount of adsorbed nitrogen gas in a relative vapour pressure of 0.05–0.3 at 77 K by Brunauer–Emmett–Teller analysis.

XP spectra were measured with a Kratos Axis Ultra spectrometer, using monochromated AlK_α_ X‐rays (*hν*=1486.6 eV) in normal emission geometry. High resolution XPS data were fitted using UNIFIT2007^51^ employing a Shirley‐type background and peaks defined by a convolution between Gaussian‐Lorentzian lineshapes, with the exception of the main C 1s line, which is fitted with the asymmetric Doniach‐Šunjić lineshape characteristic of graphitic materials.^52^ It was not possible to determine a unique value for asymmetry parameter of the Doniach‐Šunjić line and hence a value of 0.14, consistent with other nanostructured graphitic carbons, was chosen.^37^ However, similar results were obtained using asymmetries characteristic of bulk graphite.^52^ The accuracy of resulting fits was attested to by reduced *χ* ^2^ values close to 1 and minimal systematic variation in the fit residuals.

DTA and TGA measurements were carried out in air with a heating rate of 10 °C min^−1^ from 100 to 800 °C. Pure alumina was used as the reference material. The accuracy of these analyses is about 1–2 %.
